# Association between weaning stress and rumen microbiota in goat kids: evidence from granger causality and randomized controlled trial validation

**DOI:** 10.5713/ab.25.0092

**Published:** 2025-08-25

**Authors:** Ziwei Peng, Hanjie Xiao, Weiwei Yang, Tong Wang, Haojiong Xie, Hui Yan, Shoukun Ji

**Affiliations:** 1 College of Animal Science and Technology, Hebei Agricultural University, Baoding, China

**Keywords:** Microbial Maturity, Rumen Microbiota Transplantation, Weaning Stress

## Abstract

**Objective:**

Although there is some evidence linking weaning stress to altered gut flora and stunted development, the exact nature of this relationship is still up for debate.

**Methods:**

In this study, we employed Granger causality inference to compare the dynamic changes in gastrointestinal microbiota between stressed and non-stressed goat kids, and we validated our findings through a randomized controlled trial involving rumen microbiota transplantation.

**Results:**

Our findings indicated that the alpha diversity and microbial maturity of the rumen microbiota in stressed kids were significantly lower than those in non-stressed kids during the weaning period (p<0.05). Furthermore, the rumen microbiota at pre-weaning could accurately predict the body weight changes during weaning stress period (R^2^ = 0.99, root-mean-square error of prediction = 0.19). We found that the majority of high-abundance rumen bacteria (89.22%) were retained by the cultured rumen microbiota, and that transplanting this microbiota successfully changed the rumen microbiota (analysis of similarity of microbiota, p<0.05), improved growth performance (p<0.05) during weaning, and enhanced microbial maturity (p<0.05), but had no effect on the gut microbiota (p>0.05). Additionally, transplantation of the cultured rumen microbiota reduced intestinal permeability and inflammation while increasing antioxidant levels in weaned kids (p<0.05).

**Conclusion:**

These findings provide evidence for the association between rumen microbiota and weaning stress, demonstrating that manipulating rumen microbiota is an effective strategy for alleviating weaning stress in goat kids.

## INTRODUCTION

Weaning stress is a common physiological response in mammals during the weaning process, triggered by factors such as maternal separation, dietary changes, and environmental alterations [1]. This stress typically manifests within one week post-weaning, leading to reduced immunity, decreased feed intake, and increased susceptibility to diseases [2], severely stressed animals often exhibit diarrhea [3]. Studies have shown that mammals during the weaning often experience significant weight loss [4], with recovery typically occurring around 14 days post-weaning [5]. These issues increase the feeding and management costs in animal production [6].

Rumen and gut harbor a vast array of microorganisms, including bacteria, archaea, fungi, and protozoa, which play crucial roles in maintaining the gastrointestinal homeostasis [7,8]. Gastrointestinal microbial community shift during weaning has been widely observed [1,9,10], with V-shaped rumen microbiota diversity during weaning [5]. Changes in microbial composition, such as reduced Bacteroidetes and increased Firmicutes [5], as well as increased fiber-degrading bacteria [11] and increased harmful bacteria post-weaning [12]. However, the association between them remains poorly understood.

Herein, we hypothesize that gastrointestinal microbiota dysbiosis contributes to weaning stress. Previous studies indicated that Granger causality, a statistical concept based on prediction, posits that if a time series X provides statistically significant information about the future values of a time series Y, then X Granger-causes Y [13], and microbiota transplantation could provide proof-of-concept for microbiome therapeutics and causal relationship validation [14]. To investigate the association between weaning stress and gastrointestinal microbiota in goat kids, we used Granger causality analysis and rumen microbiota transplantation to address three key questions: 1) Is there a difference in the assembly process of gastrointestinal microbiota between stressed and non-stressed kids? 2) Can transplantation of gastrointestinal microbiota from non-stressed kids alleviate weaning stress in recipient (stressed) kids? If so, 3) how does the gastrointestinal microbiota affect the weaning stress?

## MATERIALS AND METHODS

### Experimental animal and feeding management

**Figure 1:**
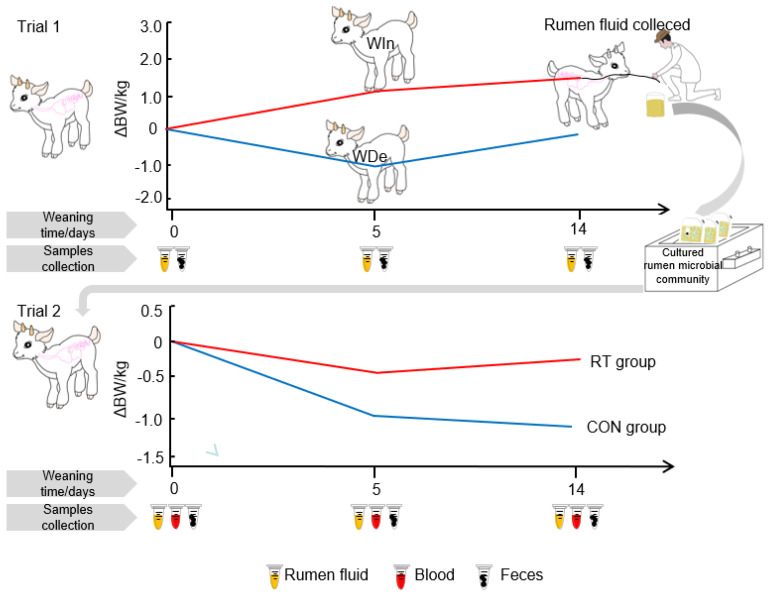
Experimental setup. In trial 1, 6 goat kids with weaning stress (WDe) and 5 goat kids with non-weaning stress (WIn) were selected based on the body weight change (ΔBW) during the weaning period, and microbiota change in rumen and gut was monitored at pre-weaning (day 0), day 5 and 14 post-weaning. Rumen microbiota from goat kids at 14 days post-weaning was collected and then cultured with *in vitro* batch fermentation to achieve a stable rumen microbial community. In trial 2, 12 pre-weaning goat kids were randomly assigned into 2 groups, with goat kids in one group receiving oral infusion with cultured rumen microbiota (RT), and kids in another group receiving an equal amount of physiological saline (CON), then the microbiota in rumen and gut as well as serum parameters were monitored at pre-weaning (day 0), day 5 and 14 post-weaning.

This study consisted of two trials (Figure 1), and male Yanshan Cashmere goat kids were used in both trials. The kids (two weeks before weaning) with similar body weights were selected (Detailed data can be found in Tables 1, 2), and when each kid weighs reached approximately 13 kg (at 65–70 days of age), weaning was performed abruptly by separating the kid from its dam.

Trial 1: 21 kids were used for comparing the gastrointestinal microbiota during the weaning period to explore the assembly process difference of gastrointestinal microbiota with different degrees of weaning stress in goat kids;

Trial 2: 12 kids were used for rumen microbiota transplantation to discover could the altered gastrointestinal microbiota inhibit the weaning stress and why does it work. In addition, 4 healthy kids at 14 days post-weaning (from Trial 1) were selected as donors for rumen fluid collection to culture the rumen microbial community, which was subsequently used for rumen microbiota transplantation.

The experimental pens were thoroughly cleaned and disinfection was carried out weekly before and during the trial. Before weaning, all kids were housed together with their dams in large pens, where they could move freely and nurse, and supplemented with basal feed (Supplement 1) with free access to feed and water; After weaning, the goat kids were transferred to experimental pens, each pen housed 5–6 kids, and basal feed was provided twice daily at 8:00 and 16:30 with about 10% feed residual. Body weights of kids were measured before the morning feeding at days 0, 5, and 14 post-weaning and individual fecal scores were recorded each day following the previous method (Supplement 2) [15].

### Group assignment of goat kids

For the trial on comparing the gastrointestinal microbiota, 21 pre-weaning goat kids were selected. The kids were assigned into 3 groups based on their weight change and fecal scores within 5 days after weaning: 1) Weaning stress group (WDe): kids with the greatest weight decrease and without diarrhea by fecal scores<2 (n = 6); 2) Non-weaning stress group (WIn): kids with body weigh increase or the least weight loss and without diarrhea by fecal scores<2 (n = 5); and 3) Intermediate group (MWs): Other kids with moderate body weight change and the fecal scores>2 (n = 10).

For the trial on rumen microbiota transplantation, 12 goat kids were randomly assigned into 2 groups at weaning. Starting on the day of weaning (day 0), kids in rumen microbiota transplantation group (RT, BW: 12.02±0.79) were orally infused with 100 mL cultured rumen microbial community twice daily (08:00 and 18:00) for 3 consecutive days, while kids in control group (CON, BW: 12.48±0.57) received 100 mL physiological saline at the same times. One kid in the RT group was diagnosed with pneumonia during the weaning period, which was removed in this trial.

### Cultured rumen microbial community

Rumen digesta from the four donor goat kids (n = 4) at 14 days post-weaning were collected via esophageal intubation. These donors were selected based on excellent health and growth performance — ensuring they were well-adapted and had recovered from weaning stress — and were thoroughly examined by an experienced veterinarian to confirm the absence of any infections or other chronic disease risk factors. The collected digesta was pooled, and the rumen microbial community was cultured by in vitro fermentation method following our previous study [16].

In brief, rumen fluid was mixed with rumen buffer (pH = 6.86) at a 1:2 volume ratio to prepare a mixed rumen inoculum. A total of 30 g of feed (Supplement 1) was weighed and added into a 1 L fermentation bag, which was then sealed and deoxygenated. Subsequently, 300 mL of the prepared mixed rumen fluid was injected into the fermentation bag using a syringe. The bag was placed in a constant-temperature water bath shaker at 39°C for anaerobic fermentation (45 rpm/min) for 24 hours.

On the second day, the 24-hour fermented rumen fluid was again mixed with rumen buffer at a 1:2 ratio, followed by the addition of feed, and fermentation was continued under the same conditions for another 48 hours. The continuous culture *in vitro* was carried out in 8 rounds. Through this process, the cultured rumen fluid required for the experiment was successfully obtained. Collecting cultured rumen fluid after the 3rd, 4th, 7th, and 8th rounds of fermentation to detect microbial composition.

### Samples collection

Rumen digesta and fecal samples from the goat kids of the two trials were collected before the morning feeding at pre-weaning (day 0), day 5, and day 14 post-weaning via esophageal intubation or rectum, respectively. To avoid saliva contamination for rumen digesta, the first 20 mL of achieved rumen digesta was discarded before each sample collection, then the rumen digesta was filtered through four layers of cheesecloth, and the filtrate was harvested as rumen fluid samples. Both the rumen fluid samples and fecal samples were transferred into a 2 mL sterile tube, which was quickly immersed in liquid nitrogen for subsequent microbial analysis.

Blood samples from the goat kids of the rumen microbiota transplantation trial were collected before the morning feeding at pre-weaning (day 0), 5, and 14 post-weaning via cervical vein, the blood was then centrifuged at 1,500×g 4°C for 5 min, the serum was transferred into a 2 mL sterile tube, which was quickly immersed in liquid nitrogen for subsequent analysis.

### Serum parameters measurement

Antioxidant indices (malondialdehyde, MDA; total antioxidant capacity, T-AOC), inflammatory factors (tumor necrosis factor-α, TNFα; interleukin-1β, IL-1β; interferon-γ, IFN-γ) and intestinal permeability indices (lipopolysaccharide, LPS; diamine oxidase, DAO) were determined by ELISA kits (the intraassay CV<15%) according to the manufacturer’s instructions (Beijing Sino-UK Institute of Biological Technology), and the absorbance was measured using a microplate reader (RT-6100; Rayto Life and Analytical Sciences); Superoxide dismutase (SOD) and glutathione peroxidase (GSH-Px) enzyme activities were measured using an biochemical analyzer (BK-280; Biobase Biodustry [Shandong]).

### High-throughput sequencing of the 16S rRNA gene

Genomic DNA was extracted from the rumen fluid and fecal samples using the E.Z.N.A. Soil DNA Kit (Omega Bio-tek), and DNA quality and concentration were assessed using the Nanodrop 2000 (Thermo Fisher Scientific). The V3–V4 region of the bacterial 16S rRNA gene was amplified using the universal primers 338F (5′-ACTCCTACGGGAGGCAG CAG-3′) and 806R (5′-GGACTACHVGGGTWTCTAAT-3′) on an ABI9700 PCR instrument (Applied Biosystems). The PCR reaction was carried out in a 25 μL mixture containing 2 μL DNA template, 1 μL forward primer (5 μM), 1 μL reverse primer (5 μM), 3 μL BSA (2 ng/μL), 12.5 μL 2× Taq PCR MasterMix, and 5.5 μL ddH_2_O. The PCR program consisted of an initial denaturation at 95°C for 5 min, followed by 28 cycles of denaturation at 95°C for 45 s, annealing at 55°C for 50 s, extension at 72°C for 45 s, a final extension at 72°C for 10 min, and a final hold at 4°C. PCR products were purified using the Agencourt AMPure XP purification kit (Beckman Coulter). Libraries were constructed using the NEB Next Ultra II DNA Library Prep Kit (New England Biolabs) and sequenced on the Illumina Novaseq 6000 platform.

### Microbial bioinformatic analysis

Raw sequencing data were assigned to individual samples based on barcodes. The sequencing data were filtered and assembled using Pear software (ver. 0.9.6), and chimeric sequences were removed, then High-quality sequences were clustered into operational taxonomic units (OTUs) using Vsearch software (ver. 2.7.1) with a sequence similarity threshold of 97%. Representative sequences were then aligned against the Silva138 database to obtain taxonomic information for each OTU. Phylogenetic trees were constructed using the online tool iTOL ( https://itol.embl.de/).

### Calculation of α and β diversity indices

The α and β diversity indices were calculated using the QIIME software (ver. 1.8.0) in conjunction with the R software (ver. 3.6.0). The Richness index, Evenness index, and Diversity index was calculated following previous studies [17–19]. In addition, the maturity was calculated using the following formula [20]: maturity = 1−(∑|Ai−Bi|)/∑(Ai+Bi), Where Ai is the relative abundance of the ith species in sample A, Bi is the relative abundance of the ith species in sample B.

### Prediction model with RandomForest algorithm

Granger causality framework [21,22]: the RandomForest model was constructed using the RandomForest package (ver. 4.7.1.1) in R software (ver. 4.3.1) to predict the growth performance of goat kids based on microbial data (ntree = 500), and feature bacteria was selected by increasing of the mean squared error (%IncMSE) with cross-validation. The accuracy of the predictive model was evaluated using R-squared (R^2^) and the root-mean-square error of prediction (RMSPE).

### Statistical analysis

All data were analyzed using R software (ver. 4.3.1). The comparison of growth performance in Trial 1 were performed by one-way ANOVA followed by Duncan’s post-hoc test for pairwise differences; The growth performance in Trial 2 and alpha diversity, microbial maturity, serum parameters between groups were analyzed with independent t-tests. PCoA analysis were performed with vegan package (ver. 2.5–6), and comparison of microbiota composition was conducted with analysis of similarity of microbiota (ANOSIM). Data were presented as means±standard error of the mean (SEM), and a p-value lower than 0.05 was considered a significant difference.

## RESULTS

### Dynamic changes of gastrointestinal bacterial community during the weaning period of goat kids

To investigate the dynamic change of gastrointestinal microbiota during weaning period, 21 goat kids were assigned into 3 groups based on their body weight change and fecal scores after weaning: the weaning stress group (WDe), non-weaning stress group (WIn), and intermediate group (MWs; Figure 1). We observed that the body weight of the WDe group decreased and was significantly lower than that of the WIn group at 5 days post-weaning (p<0.01), but during 5 to 14 days post-weaning, the body weight of kids in the WIn group and WDe group increased, indicating a recovery from weaning stress at 14 days post-weaning (Table 1).

**Figure 2:**
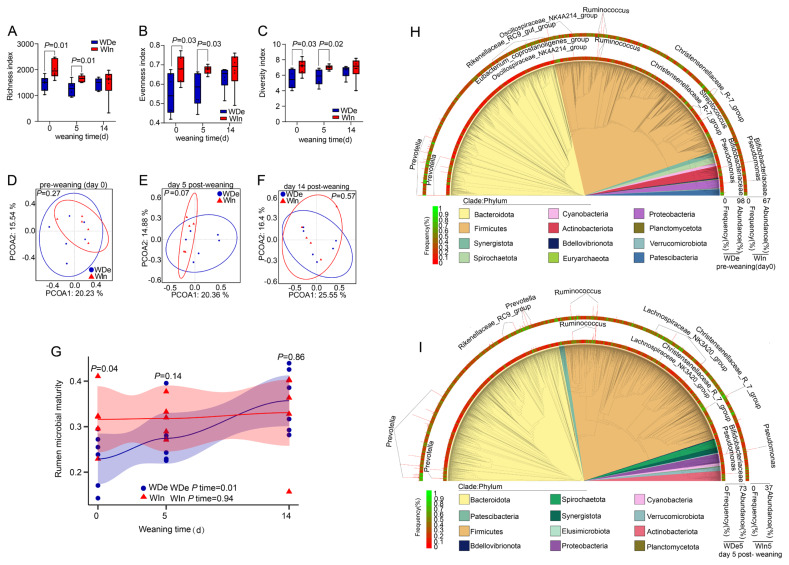
Dynamic changes in body weight and ruminal bacterial communities during the weaning stress period. (A) Richness index, (B) Evenness index, and (C) Diversity index of rumen microbiota between groups. Principal coordinate analysis (PCoA) of rumen microbiota at the OTU level in goat kids at pre-weaning (0 days; D), 5 days (E), and 14 days (F) post-weaning. (G) The maturity of rumen microbiota in the WDe and WIn groups. Phylogenetic tree of rumen microbiota at pre-weaning (0 days; H) and 5 days post-weaning (I). WDe is the weaning stress group with goat kids having the greatest weight decrease and without diarrhea, Win is the non-weaning stress group with goat kids having a body weight increase or the least weight loss and without diarrhea, and MWs is the intermediate group. Detailed information on high-abundance and high-frequency rumen bacteria in Figures 2H, 2I is shown in Supplement 5. OTU, operational taxonomic unit.

We further explored the dynamic change of the rumen and gut bacterial community during the weaning period between the WIn group and WDe group using high-throughput sequencing, and found that the Richness, Diversity, and Evenness indices of the rumen microbiota in the WIn group were significantly higher than those in the WDe group at pre-weaning (day 0) and day 5 post-weaning (p<0.05), but there were no significant differences between groups at day 14 post-weaning (p>0.05; Figures 2A–2C), meanwhile, we did not detect any differences in gut microbiota between groups (p>0.05; Supplements 3A–3C). No significant differences were detected in either the rumen or gut microbial community composition between the WIn and WDe groups during the weaning stress period with Bray-Curtis distance (ANOSIM, p>0.05; Figures 2D–2F; Supplements 3D–3F), but there was a trend to be different in rumen microbial community at 5 days post-weaning (ANOSIM, p = 0.07; Figure 2E).

We hypothesized that the rumen and gut microbiota at 14 days post-weaning represented a mature and stable condition, representative rumen and gut communities were constructed by calculating mean abundance of individual sample at 14 days post-weaning, respectively (Supplements 4A, 4B), and the maturity of the rumen and gut microbiota was calculated based on the Bray-Curtis similarity, we observed the maturity of the rumen bacterial community in the WIn group at pre-weaning (day 0) was significantly higher than that in the WDe group (p<0.05), and the maturity in the WDe group gradually increased (ptime<0.05), while that of the WIn group did not change and kept stable during the weaning period (ptime> 0.05; Figure 2G). In addition, the maturity of gut microbiota was not affected (p>0.05) and kept stable for both groups during the weaning period (ptime>0.05; Supplement 3G).

We then further explored the rumen bacterial composition of goat kids. At pre-weaning (day 0), a huge difference in high-abundance (relative abundance≥5%) and high-frequency (appearance in frequency = 100%) bacteria (OTU level) between the two groups was observed. Specifically, there were 5 high-abundance bacteria in the WIn group and 17 in the WDe group, 275 high-frequency bacteria in WIn group and 170 in the WDe group, with the number of shared bacteria only accounted for ~22% (Figure 2H, Supplement 5). Similarly, at day 5 post-weaning, there were 8 high-abundance bacteria in the WIn group and 12 in the WDe group, with the number of shared bacteria accounted for ~10%, meanwhile, there were 348 high-frequency bacteria in the WIn group, and 179 in the WDe group, with the number of shared bacteria only accounted for 22.2% (Figure 2I, Supplement 5). These observations indicated that the rumen bacterial community of goat kids with weaning stress and non-weaning stress showed differences at both pre-weaning (day 0) and day 5 post-weaning.

### Relationship between weaning stress and rumen bacteria of goat kids

**Figure 3:**
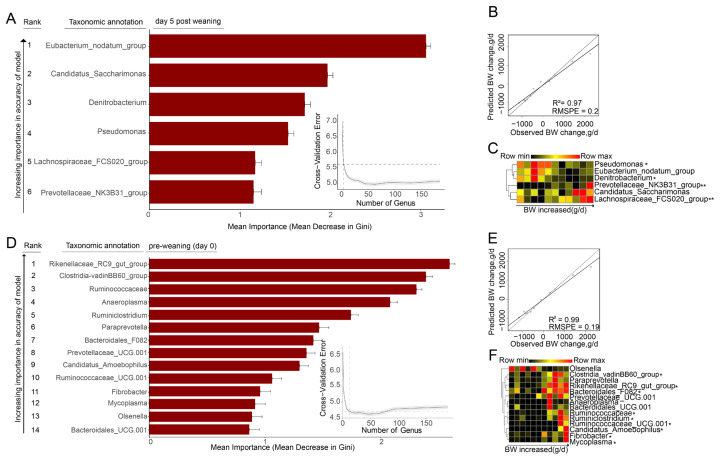
Correlation between degree of weaning stress (body weight change in 5 days post-weaning) and rumen bacteria. (A) Signature bacteria associated with body weight change (ΔBW_0–5_) based on rumen bacterial composition at day 5 post-weaning detected with the Random Forest model. (B) Accuracy assessment of the prediction of body weight change of goat kids by the detected signature bacteria at day 5 post-weaning. (C) Correlation of the relative abundance of the detected signature bacteria at day 5 post-weaning with body weight change. (D) Signature bacteria associated with body weight change (ΔBW_0–5_) based on rumen bacteria composition at pre-weaning (day 0) detected with the Random Forest model. (E) Accuracy assessment of the prediction of body weight change of goat kids by the detected signature bacteria at pre-weaning (day 0). (F) Correlation of the relative abundance of the detected signature bacteria at pre-weaning (day 0) with body weight change. ΔBW: We define body weight change (ΔBW) as the difference between the weight at a given day after weaning and Day 0 (pre-weaning); “*” and “**” indicates significant difference at p<0.05 and p<0.01, respectively.

To further explore the relationship between weaning stress and rumen bacteria, we first used the RandomForest machine learning algorithm to predict the body weight change (ΔBW_0–5_) of goat kids using bacterial composition of day 5 post-weaning at the genus level, and a total of 6 signature bacteria related to body weight changes were selected through cross-validation curves (Figure 3A), we demonstrated that these 6 signature bacteria could accurately predict the body weight change of kids (R^2^ = 0.97, RMSPE = 0.22; Figure 3B). Furthermore, the relative abundances of *Lachnospiraceae_FCS020_group* and *Prevotellaceae_NK3B31_group* was positively correlated (p<0.05), while those of *Denitrobacterium* and *Pseudomonas* were negatively correlated (p<0.05) with body weight changes of kids (Figure 3C). This result indicated that the degree of weaning stress of kids was correlated with the rumen bacterial composition at 5 days post-weaning.

We then used the rumen bacterial composition at pre-weaning (day 0) of goat kids to predict body weight changes (ΔBW_0–5_), a total of 14 signature bacteria related to weight changes were selected through cross-validation curves (Figure 3D), and these 14 signature bacteria could also accurately predict the body weight change of kids at day 5 post-weaning (R^2^ = 0.99, RMSPE = 0.19; Figure 3E). *Rikenellaceae_RC9_gut_group*, *Mycoplasma*, *Ruminiclostridium*, *Candidatus_Amoebophilus*, *Bacteroidales_F082*, *Fibrobacter*, *Ruminococcaceae*, *Ruminococcaceae_UCG-001*, and *Clostridia_vadinBB60_group* were positively correlated with the body weight changes of kids (p<0.05; Figure 3F).

### The effects of cultured rumen bacterial community on gastrointestinal microbiota and growth performance of weaning goat kids

**Figure 4:**
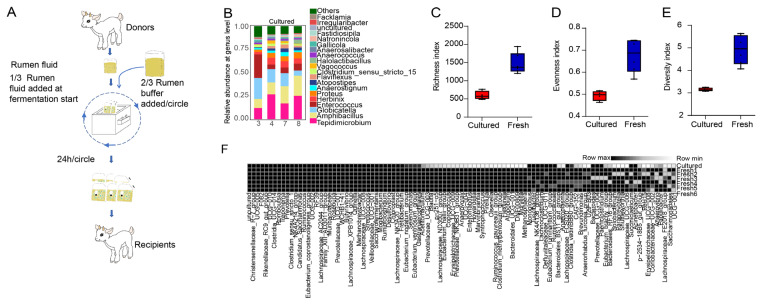
Composition of the cultured rumen bacterial community and fresh rumen bacterial community. (A) The cultivation process of rumen microbiota. (B) Collecting cultured rumen fluid after the 3rd, 4th, 7th, and 8th rounds of fermentation, and compare the bacterial community composition at the genus level. Microbial richness (C), evenness (D), and diversity (E) indices for the cultured and fresh rumen bacterial community. (F) Visual comparison of relative abundance of the core bacterial genera (frequency of appearance in fresh ruminal bacterial community = 100%) in the cultured and fresh rumen fluid.

To circumvent potential risks and uncertainties associated with directly using fresh rumen digesta as the microbial source, the rumen microbial community was cultured and enriched with in vitro fermentation for 8 days. The cultivation process of rumen microbiota is presented in Figure 4A. We observed the rumen microbiota was stable for 3 to 8 days during culture (Figure 4B), and the Richness, Evenness, and Diversity indices of cultured rumen microbiota decreased compared with the fresh rumen fluid (Figures 4C–4E), but the ruminal core bacteria (frequency of appearance in fresh ruminal bacterial community = 100%) at genus level remained with high proportion at 89.22% (Figure 4F).

**Figure 5:**
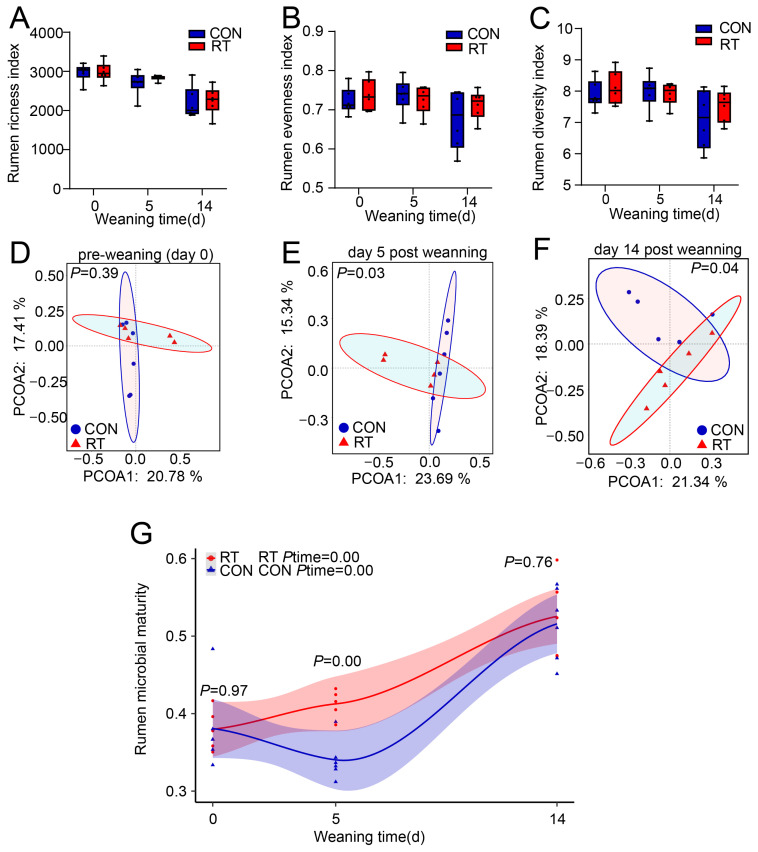
The effects of cultured rumen microbiota transplantation on rumen microbiota and growth performance of weaning goat kids. (A) The effect of cultured rumen microbiota transplantation on the growth performance of goat kids. Richness index (B), evenness index (C), and diversity index (D) of rumen microbiota between groups. Principal coordinate analysis (PCoA) of rumen microbiota at the OTU level at pre-weaning (0 days, E), 5 days (F), and 14 days (G) post-weaning. (H) The maturity of rumen microbiota in the RT and CON groups. RT was the rumen microbiota transplantation group; CON was the control group. OTU, operational taxonomic unit.

We then performed rumen microbiota transplantation with the cultured rumen microbiota for three consecutive days, starting on the day of weaning (day 0). We observed that the body weight loss of goat kids receiving cultured rumen microbiota (RT) was lower than that of goat kids receiving physiological saline (CON) at both 5 and 14 days after weaning (Table 2). The richness, evenness, and diversity of rumen and gut microbiota were not affected by the rumen microbiota transplantation (p>0.05; Figures 5A–5C; Supplements 6A–6C), and both rumen bacterial composition and gut bacterial composition was similar at pre-weaning (day 0; ANOSIM, p>0.05; Figure 5D; Supplement 6D), but the rumen bacterial composition at 5 and 14 days post-weaning was significantly affected (ANOSIM, p<0.05; Figures 5E, 5F), and no significant difference was observed in the gut bacterial composition between kids in CON and RT groups after weaning (ANOSIM, p>0.05; Supplements 6E, 6F). In addition, we also observed the maturity of the rumen bacterial community increased during the weaning period (ptime<0.05), and the maturity of the RT group at day 5 post-weaning was significantly higher than that of the CON group (p<0.05 Figure 5G), meanwhile, the maturity of the gut bacterial community was stable during the weaning period (ptime>0.05) and no difference was observed between the two groups (p>0.05; Supplement 6G).

### The effects of cultured rumen bacterial community on serum parameters of weaning goat kids

**Figure 6:**
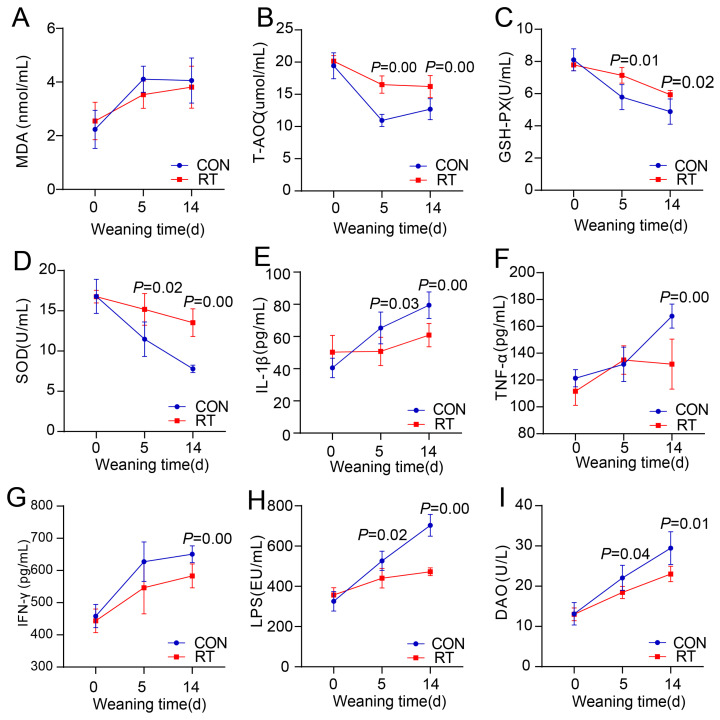
The effects of cultured rumen microbiota transplantation on serum parameters of weaning goat kids. The effects of cultured rumen bacterial communities on antioxidant indicators MDA (A), T-AOC (B), GSH-PX (C), SOD (D); inflammatory indicators IL-1β (E), TNF-α (F), IFN-γ (G); and intestinal permeability indicators LPS (H), DAO (I) in weaning goat kids. RT was the rumen microbiota transplantation group; CON was the control group. MDA, malondialdehyde; T-AOC, total antioxidant capacity; GSH-PX, glutathione peroxidase; SOD, superoxide dismutase; IL-1β, interleukin-1β; TNFα, tumor necrosis factor-α; IFN-γ, interferon-γ; LPS, lipopolysaccharide; DAO, diamine oxidase.

To investigate why transplantation of the cultured rumen microbiota could inhibit weaning stress in goat kids, we further analyzed the serum parameters of kids in CON and RT groups. We observed that MDA content was not different between the two groups (Figure 6A), but the serum T-AOC, GSH-PX, and SOD content of kids in the RT group was significantly higher than that in the CON group at day 5 and 14 post-weaning (Figures 6B–6D), which represented an increase in antioxidant by transplantation of cultured rumen microbiota; We also observed the serum IL-1β content in RT group was significantly lower at day 5 and 14 post-weaning (Figure 6E), serum TNF-α and IFN-γ content was lower at day 14 post-weaning than that in CON group (Figures 6E–6G); Intestinal permeability associated index such as serum LPS and DAO content in RT group was lower at both day 5 and 14 post-weaning (Figures 6H, 6I), indicated an increase in gastrointestinal epithelial integrity by transplantation of cultured rumen microbiota. In addition, the serum parameters were similar at pre-weaning (day 0, p>0.05), but significantly changed at the post-weaning period between groups (day 5 and 14, p<0.05), which also demonstrated a significant effect of transplantation of cultured rumen microbiota on the serum parameters during the weaning period.

## DISCUSSION

Weaning stress is a common phenomenon during the weaning period of mammals, primarily characterized by a decreased growth rate or a loss of body weight [4], which might be potentially caused by the un-adaptation of the gastrointestinal microbiota [12]. In this study, two animal groups were compared, with the goat kids that continually increased in body weight after weaning as the non-stress group (WIn) and goat kids that decreased body weight after weaning as the weaning stress group (WDe). We found that the body weight of goat kids in the WDe group was lowest at day 5, and returned to the pre-weaning level at day 14 post-weaning. Meanwhile, with microbial diversity analysis, we also found that at pre-weaning (day 0) and day 5 post-weaning, the richness, evenness, and diversity of the rumen microbiota in the WIn group were significantly higher than those in the WDe group, and there was no significant difference between the two groups at day 14 post-weaning. These findings were consistent with previous study [5], which reported that the weaning stress usually happened in ~7 days post-weaning and recovered in ~14 days post-weaning.

Therefore, we hypothesized that the gastrointestinal bacterial community had recovered from the un-adaptation situation by day 14 post-weaning, and a reference bacteria community was constructed to calculate the maturity of the gastrointestinal microbiota following previous studies [23], the maturity index reflects the similarity of the gastrointestinal microbiota during weaning to the reference bacterial community [24]. Our results indicated that the maturity of the rumen bacterial community in goat kids with non-weaning stress remained stable during weaning, while that in kids with weaning stress significantly decreased at pre-weaning (day 0) and gradually increased after weaning, reaching a similar condition with the non-stress group by day 14 post-weaning, but the gut microbiota was not affected. These results suggested that kids with weaning stress suffered from weight loss after weaning, accompanied by a decrease in rumen microbial maturity.

The composition of rumen microbiota determines their function and may potentially affect weaning stress [25]. We found that the predominant rumen bacteria at phylum were Firmicutes, Bacteroidetes, Actinobacteria, and Proteobacteria, which was consistent with previous studies [17,18,26], but the shared high-abundance and high-frequency bacteria between goat kids with weaning stress and non-weaning stress were less than 23%. We also confirmed the rumen microbiota at both pre-weaning (day 0) and post-weaning (day 5) as a predictor that can accurately predict the weight change of kids after weaning with the RandomForest algorithm. An accurate prediction always revealed the correlation between predictor variables and response variables [19]. Here, we found that a significant difference in the rumen bacterial composition of stressed & non-stressed kids, which might be the potential reason for the weaning stress in kids [20]. Importantly, the state of the rumen microbiota before weaning determines the severity of weaning stress experienced by the kids after weaning. This conclusion is supported by our observations that the rumen microbiota of weaning stress kids had significantly different from that of non-stress and recovered kids, with these microbial changes occurring before the onset of weaning stress. These observations suggested that the rumen microbiota was a Granger causality of weaning stress [13]. This supports the pre-emptive acclimation theory [27], which proposes that pre-adapting the rumen microbiota to post-weaning condition can effectively reduce weaning stress in kids.

Rumen microbiota transplantation is an efficient strategy to manipulate rumen microbiota [14], which provides the necessary proof-of-concept for causal relationship validation. To circumvent the associated risks and uncertainties of rumen microbiota from fresh rumen fluid, we first cultured rumen microbiota by a continuous batch culture method, and demonstrated the cultured rumen microbiota lost some bacteria (diversity decreased), but most (89.22%) of the core bacteria remained, this result was similar with our previous finding [16]. Subsequent transplantation of this cultured rumen microbiota significantly enhanced the growth performance and increased the maturity of the rumen microbiota in goat kids during the weaning period. These findings demonstrated that cultured rumen microbiota transplants could also benefit animals, aligning with observations from studies using fresh rumen microbiota or frozen rumen microbiota for transplantation [28–30].

We observed that transplantation of cultured rumen microbiota efficiently enhanced the maturity of the rumen microbiota but failed to regulate the gut microbiota. This result might be due to two reasons: 1) the transplanted microbiota was originally from the rumen, and these bacteria suited the rumen environment, although some ruminal microbes might pass through the abomasum into the intestine [31], the number might be limited, and their adaptability in the intestine was another barrier to their colonization [32]; 2) upon our investigation, the rumen microbiota was in a state of flux while the gut microbiota was stable during the weaning period, a substantial number of ecological niches become vacant in a dynamic condition, creating a conducive environment for the colonization of the introduced bacteria [33], and conversely, a stable and mature gastrointestinal microbiota can form a resistance to the colonization of the introduced bacteria [34].

To investigate why transplantation of cultured rumen microbiota affected the weaning stress, we also measured serum parameters associated with intestinal permeability, inflammation, and antioxidants, which have been proven to be sensitive indicators reflecting stress in animals [35,36], and observed that the antioxidant increased, intestinal permeability and inflammation decreased after transplanting the cultured rumen microbiota. These benefits on serum parameters were also observed in some previous studies [37,38]. Taken together with our finding that the maturity of rumen microbiota increased by transplantation of cultured rumen microbiota, we could infer that the direct effect of cultured rumen microbiota transplantation might be manipulating the rumen microbiota from a dysbiosis condition to a eubiosis condition [28], this eubiosis condition of rumen microbiota manifested as an increased adaptation to the post-weaning rumen function [39]. Then the eubiosis condition of rumen microbiota might be helpful for the rumen epithelial integrity as our observations that the amount of LPS and DAO escaped from rumen into blood decreased [40]. With rumen epithelial integrity increase, the invading macromolecules and pathogenic bacteria from the rumen into the bloodstream might also decrease, which contributed to the inflammation decrease and antioxidant increase [41]. Although the gut microbiota composition was not significantly altered by rumen microbiota transplantation, we acknowledge that the gut is the primary site for LPS absorption into the bloodstream [42]. It is possible that changes in the rumen microbial composition indirectly affected intestinal barrier function or LPS translocation through immune or metabolic cross-talk between the rumen and the intestine, even in the absence of major compositional changes in gut microbiota.

Not consistent with our findings in a previous study, which observed transplantation of fresh rumen microbiota from 3-months and 1-year-old lambs accelerated the maturity of rumen microbiota but impaired the gastrointestinal health and growth performance [43]. In that study, the recipient animals were thirty-eight pre-weaning Hu breed lambs (age = 55 days old). This contrary observation might be mainly because of the different donor situations, and a developmentally mismatched of the microbial community caused by transplantation from 3-months and 1-year-old animals was also a kind of dysbiosis [44], which might also be harmful to the young host. Previous studies demonstrated that manipulating the rumen microbiota from a dysbiosis condition to a eubiosis condition would benefit animals [39,44], which meant that modulating the intestinal microbiota to a functionally optimal and adapted state for the post-weaning period, instead of just augmenting the maturity of the microbiota, was crucial for animals to derive benefits.

It is important to note that the present study has some limitations. Firstly, we performed two longitudinal studies with 136 samples using 16S rRNA gene sequencing without metagenomic sequencing and metabolomic analysis, which limited our ability to provide detailed information about specific microbial species and their functional roles [45]. Future investigations combining metagenomic, metabolomic, and functional assays will be essential to fully unravel the underlying mechanisms. Despite these constraints, we observed a stable trend in temporal data, which was also corroborated by the cross-validation between the two longitudinal studies, future studies with larger cohorts and comprehensive genomic analyses may further strengthen our understanding; However, they are unlikely to overturn the primary conclusions of the present study. Secondly, the present study confirmed the efficacy of the cultured rumen microbiota in regulating weaning stress in kids, however, there are still no standardized protocols for the rumen microbiota amplification *in vitro* to our knowledge, further systematic and in-depth investigation on it might be helpful to establish its validity and applicability in microbiota transplantation practices.

## CONCLUSION

The diversity, maturity, and composition of rumen microbiota were associated with the degree of weaning stress in kids, and the change of rumen microbiota happened before weaning. Transplantation of cultured rumen microbiota efficiently enhanced the maturity of rumen microbiota and reduced weaning stress in kids, these might be due to the rumen epithelial integrity increase, inflammation decrease, and antioxidant increase. In addition, we did not detect any association between the gut microbiota and the weaning stress in kids. These findings provided evidence for the association between weaning stress and rumen microbiota in goat kids, proposed an alternative method for regulating the rumen microbiota, and demonstrated that manipulating rumen microbiota was an efficient way of alleviating weaning stress in kids.

## Figures and Tables

**Table 1:** Dynamic changes in body weight during the weaning stress period

Items	WDe	WIn	MWs	p-value
Body weight (kg)				
Day 0	14.02±0.29	13.63±0.90	14.41±1.22	0.24
Day 5	13.23±0.27	14.51±1.21	14.32±1.20	0.57
Day 14	13.82±0.31	14.72±1.25	-	0.36
Body weight change/ΔBW (kg)				
ΔBW_0–5_	−0.78±0.21	0.88±0.42	−0.09±0.07	0.00
ΔBW_0–14_	−0.20±0.60	1.09±0.56	-	0.05

Values are presented as mean±standard error of the means.

ΔBW: We define body weight change (ΔBW) as the difference between the weight at a given day after weaning and Day 0 (pre-weaning). Thus, ΔBW_0–5_ = BW_5_–BW_0_; ΔBW_0–14_ = BW_14_–BW_0_.

WDe is the weaning stress group with body weight decreased of goat kids during weaning period, WIn is the non-weaning stress group with body weight increased, and MWs is the intermediate group.

**Table 2:** Effects of rumen microbiota transplantation on growth performance of weaned goat kids

Items	RT	CON	p-value
Body weight (kg)			
Day 0	12.02±0.79	12.48±0.57	0.64
Day 5	11.56±0.84	11.66±0.58	0.92
Day 14	11.86±0.74	11.43±0.56	0.65
Body weight change/ΔBW (kg)			
ΔBW_0–5_	−0.46±0.13	−0.83±0.06	0.02
ΔBW_0–14_	−0.16±0.11	−1.05±0.18	0.00

Values are presented as mean±standard error of the means.

ΔBW: We define body weight change (ΔBW) as the difference between the weight at a given day after weaning and Day 0 (pre-weaning). Thus, ΔBW_0–5_ = BW_5_–BW_0_; ΔBW_0–14_–BW_14_–BW_0_.

RT (rumen microbiota transplantation group): oral administration of 100 mL of cultured rumen microbiota.

CON (control group): oral administration of 100 mL of normal saline.

## SUPPLEMENTARY MATERIAL

**Supplement 1.** Composition and nutrition content of the basal diet.

**Supplement 2.** Fecal samples score of goat kids.

**Supplement 3.** Dynamic changes in intestinal bacterial communities during the weaning stress period.

**Supplement 4.** The representative rumen and gut bacterial community based on Bray-Curtis distance.

**Supplement 5.** Rumen microbiome of weaned stress goat kids at 0 and 5 days of weaning.

**Supplement 6.** The effect of cultured rumen microbiota transplantation on the intestinal bacterial communities of goat kids.
