# Supplementary material for: Association between weaning stress and rumen microbiota in goat kids: evidence from granger causality and randomized controlled trial validation
Source: Anim Biosci. 2025 Aug 25;39(1):250092. doi: 10.5713/ab.25.0092 (PMC12754500; doi:10.5713/ab.25.0092)
Supplement: Supplementary file 1 [file ab-25-0092-Supplementary-1.pdf]

Supplement 1. Composition and nutrition content of the basal diet.

Items	Content	Nutrition content	Content
Corn	62	Metabolic energy (ME), MJ/kg[2)]	11.82
Soybean meal	19	Crude protein (CP), %	17.31
Soybean	5	Neutral detergent fiber (NDF), %	10.61
Wheat bran	5	Acid detergent fiber (ADF), %	4.38
Milk powder	4	Calcium (Ca), %	1.12
Premix[1)]	4	Phosphorus (P), %	0.48
Sodium bicarbonate	0.5		
Salt	0.5		
Total	100		

[1)] The premix provided per kilogram of diet with VA 2400IU, VD3 500IU, VE 40IU, Cu 10mg, Zn 30mg, Fe 50mg, Mn 40mg, Se 0.3mg, I 2mg, Co 0.3mg.

[2)] The metabolizable energy was a calculated value, while all other nutrition contents were measured values.
