# Supplementary material for: Association between weaning stress and rumen microbiota in goat kids: evidence from granger causality and randomized controlled trial validation
Source: Anim Biosci. 2025 Aug 25;39(1):250092. doi: 10.5713/ab.25.0092 (PMC12754500; doi:10.5713/ab.25.0092)
Supplement: Supplementary file 2 [file ab-25-0092-Supplementary-2.pdf]

Supplement 2. Fecal samples score of goat kids.

Score	Trait
1	hard firm feces (health); without fetid odor
2	slightly soft feces (healthy); without fetid odor
3	soft, and partially formed feces; with a light fetid odor
4	loose, semi-liquid feces (diarrhea); with a distinct fetid odor
5	watery, mucous-like feces (severe diarrhea); with a pungent fetid odor

Reference: (Liu et.al 2021).
