# Supplementary material for: Association between weaning stress and rumen microbiota in goat kids: evidence from granger causality and randomized controlled trial validation
Source: Anim Biosci. 2025 Aug 25;39(1):250092. doi: 10.5713/ab.25.0092 (PMC12754500; doi:10.5713/ab.25.0092)
Supplement: Supplementary file 3 [file ab-25-0092-Supplementary-3.pdf]

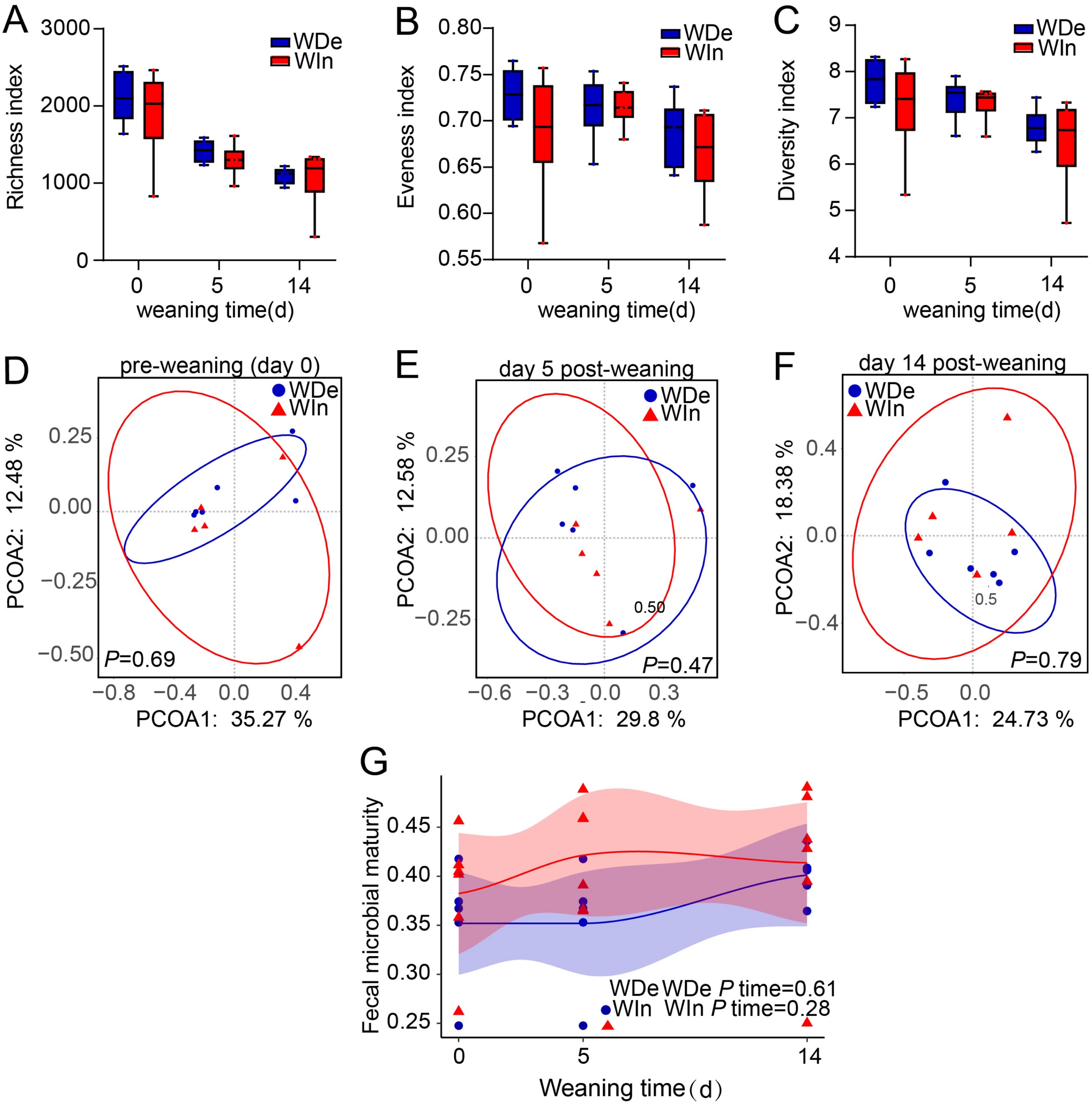

Supplement 3. Dynamic changes in intestinal bacterial communities during the weaning stress period. Richness index (A), Evenness index (B), and Diversity index (C) of intestinal microbiota between groups. Principal coordinate analysis (PCoA) of gut microbiota at the OTU level in goat kids at pre-weaning (0 days; D), 5 days (E), and 14 days (F) post-weaning. (G) The maturity of gut microbiota in the WDe and WIn groups.
