# Supplementary material for: Association between weaning stress and rumen microbiota in goat kids: evidence from granger causality and randomized controlled trial validation
Source: Anim Biosci. 2025 Aug 25;39(1):250092. doi: 10.5713/ab.25.0092 (PMC12754500; doi:10.5713/ab.25.0092)
Supplement: Supplementary file 4 [file ab-25-0092-Supplementary-4.pdf]

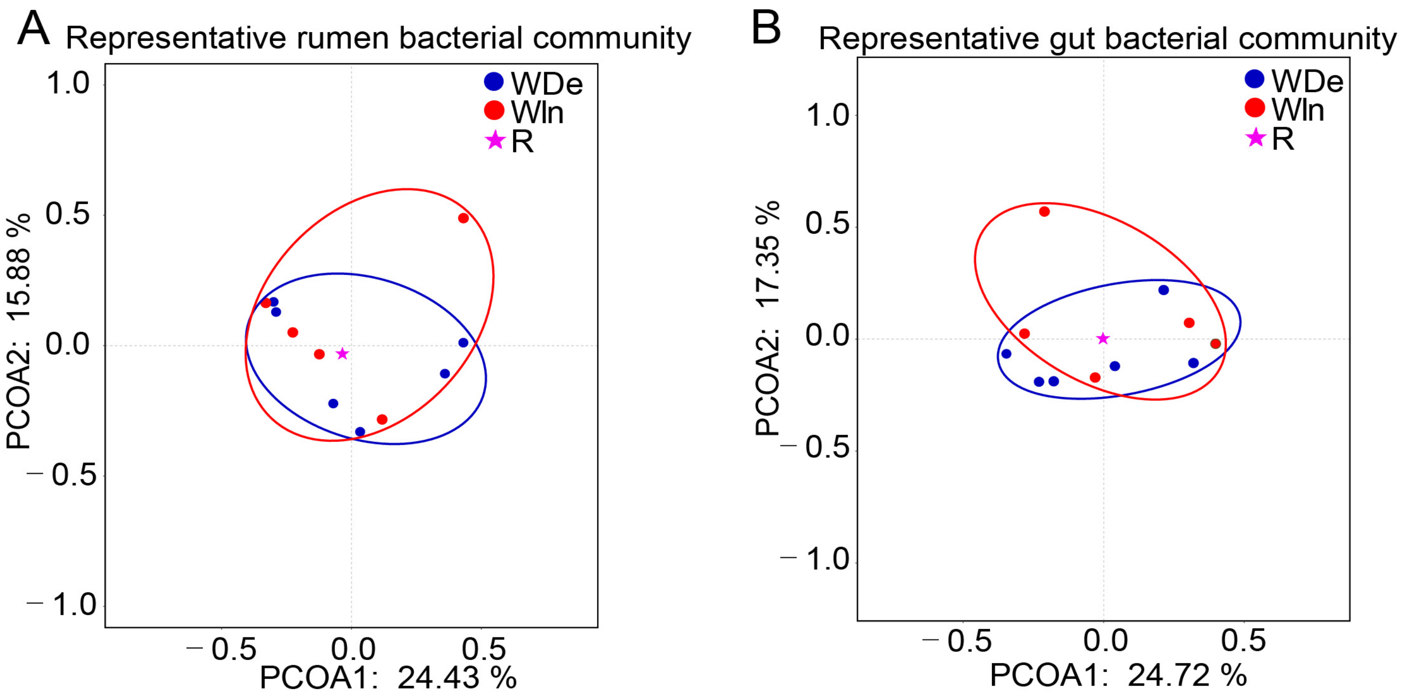

Supplement 4. The representative rumen and gut bacterial community based on Bray-Curtis distance. (A) rumen bacterial community. (B) gut bacterial community. WDe: Intense weaning stress group; WIn: Non-intense weaning stress group; R: The representative rumen and gut bacterial community at 14 days post-weaning.
