# Supplementary material for: Association between weaning stress and rumen microbiota in goat kids: evidence from granger causality and randomized controlled trial validation
Source: Anim Biosci. 2025 Aug 25;39(1):250092. doi: 10.5713/ab.25.0092 (PMC12754500; doi:10.5713/ab.25.0092)
Supplement: Supplementary file 5 [file ab-25-0092-Supplementary-5.pdf]

Supplement 5. Rumen microbiome of weaned stress goat kids at 0 and 5 days of weaning.

WIn			WDe			WIn			WDe		
pre-weaning (day 0)						5 days after weaning					
ID	Frequency (%)	Abundance	ID	Frequency (%)	Abundance	ID	Frequency (%)	Abundance	ID	Frequency (%)	Abundance
OTU_1001	100	0.000683299	OTU_704	100	0.098032339	OTU_30	100	0.020037106	OTU_30	100	0.073295172
OTU_1002	100	0.000864292	OTU_4377	100	0.033802435	OTU_4377	100	0.022896964	OTU_4377	100	0.066628203
OTU_1006	100	0.000434413	OTU_4414	100	0.076715387	OTU_704	100	0.030168786	OTU_704	100	0.058731919
OTU_1011	100	0.000122162	OTU_1106	100	0.053867441	OTU_643	100	0.010557039	OTU_643	100	0.072533448
OTU_1018	100	0.000153853	OTU_1113	100	0.04211352	OTU_4390	100	0.009099959	OTU_4390	100	0.053426249
OTU_1019	100	0.000597312	OTU_3660	100	0.030404845	OTU_3660	100	0.020448889	OTU_3660	100	0.030042838
OTU_1023	100	0.001307751	OTU_643	100	0.035755766	OTU_759	100	0.034549075	OTU_3328	100	0.01517037
OTU_1026	100	0.000171963	OTU_30	100	0.027199572	OTU_1130	100	0.037223404	OTU_3863	100	0.015743548
OTU_1035	100	0.000375567	OTU_3577	100	0.029096339	OTU_3328	100	0.016598036	OTU_3662	100	0.017798694
OTU_1059	100	0.000597317	OTU_3662	100	0.025069008	OTU_3863	100	0.012018643	OTU_4843	100	0.000599579
OTU_1083	100	0.000257925	OTU_3661	100	0.02764831	OTU_3662	100	0.008163265	OTU_764	100	0.002420924

OTU_1096	100	0.004421015	OTU_3863	100	0.00982322	OTU_926	100	0.023159419	OTU_777	100	0.004404431
OTU_110	100	0.00023982	OTU_63	100	0.019782192	OTU_946	100	0.011783339	OTU_63	100	0.011557838
OTU_1106	100	0.027218421	OTU_3328	100	0.006976183	OTU_725	100	0.016335576	OTU_3661	100	0.01061134
OTU_1108	100	9.50E-05	OTU_4324	100	0.00974026	OTU_4843	100	0.018955613	OTU_4883	100	0.01123354
OTU_1110	100	0.001353002	OTU_4619	100	0.009857166	OTU_764	100	0.01657089	OTU_874	100	0.001293422
OTU_1111	100	0.000230784	OTU_1173	100	0.008492089	OTU_777	100	0.012493778	OTU_3960	100	0.005958037
OTU_1113	100	0.02199647	OTU_5508	100	0.007805783	OTU_63	100	0.003792027	OTU_5308	100	0.003476779
OTU_1130	100	0.011887416	OTU_759	100	0.001406545	OTU_3661	100	0.004846373	OTU_825	100	0.005286815
OTU_117	100	0.001022666	OTU_777	100	0.001383924	OTU_1113	100	0.001461605	OTU_4564	100	0.004223419
OTU_1173	100	0.002778406	OTU_1204	100	0.004238502	OTU_4883	100	0.002144893	OTU_4414	100	0.004483612
OTU_118	100	0.000285076	OTU_3960	100	0.001847746	OTU_895	100	0.009140685	OTU_2102	100	0.001806266
OTU_1180	100	0.000171973	OTU_4380	100	0.004611822	OTU_874	100	0.011294629	OTU_1173	100	0.00070516
OTU_1204	100	0.004018281	OTU_110	100	0.006429405	OTU_938	100	0.003950405	OTU_925	100	0.00157624
OTU_123	100	0.000642553	OTU_4319	100	0.002839495	OTU_3960	100	0.004697045	OTU_4380	100	0.003910433
OTU_1257	100	0.000719489	OTU_764	100	0.001889226	OTU_1106	100	0.001303221	OTU_4324	100	0.003529571
OTU_1261	100	0.000294131	OTU_5322	100	0.004494924	OTU_1884	100	0.007158695	OTU_694	100	0.00549044

OTU_1267	100	0.000126702	OTU_895	100	0.001662971	OTU_825	100	0.003656279	OTU_4329	100	0.003525801
OTU_127	100	0.000407258	OTU_954	100	0.00369172	OTU_4564	100	0.004837323	OTU_1204	100	0.003446612
OTU_1282	100	0.000149318	OTU_825	100	0.000625975	OTU_4414	100	0.004018281	OTU_3681	100	0.00435163
OTU_1297	100	0.004918784	OTU_4214	100	0.002439774	OTU_2102	100	0.007136079	OTU_4319	100	0.003020499
OTU_1301	100	0.000104082	OTU_4883	100	0.000889943	OTU_1173	100	0.008041088	OTU_3579	100	0.004408189
OTU_1302	100	4.53E-05	OTU_1831	100	0.00190808	OTU_925	100	0.006090773	OTU_4619	100	0.001406549
OTU_1317	100	0.000248885	OTU_4564	100	0.002899825	OTU_937	100	0.001420888	OTU_4970	100	0.003371197
OTU_1334	100	0.000787361	OTU_4625	100	0.001640342	OTU_4380	100	0.002506901	OTU_4073	100	0.003752055
OTU_1353	100	0.00052039	OTU_4413	100	0.004065041	OTU_952	100	0.003063487	OTU_4625	100	0.000656147
OTU_1356	100	0.001276071	OTU_5348	100	0.002469946	OTU_4324	100	0.002629078	OTU_4316	100	0.002243691
OTU_1358	100	8.60E-05	OTU_3579	100	0.003744521	OTU_934	100	0.004140469	OTU_5348	100	0.002002353
OTU_1387	100	0.000312231	OTU_4314	100	0.00035069	OTU_2783	100	0.001968415	OTU_5322	100	0.002560448
OTU_1390	100	0.000108627	OTU_1309	100	0.000320523	OTU_4329	100	0.002172044	OTU_3696	100	0.002277625
OTU_1416	100	0.000131222	OTU_5333	100	0.002835732	OTU_1204	100	0.002181094	OTU_6034	100	0.002492571
OTU_1420	100	0.000167413	OTU_4843	100	0.000448738	OTU_3681	100	0.000850717	OTU_4393	100	0.002733914
OTU_1444	100	0.000153868	OTU_4313	100	0.002439786	OTU_4319	100	0.00244355	OTU_1831	100	0.001176524

OTU_1474	100	0.000235294	OTU_694	100	0.002405841	OTU_3579	100	0.000475135	OTU_4317	100	0.002748993
OTU_1491	100	0.000556591	OTU_3724	100	0.001708222	OTU_1428	100	0.005122413	OTU_5650	100	0.002360585
OTU_15	100	0.000665188	OTU_3825	100	0.002217286	OTU_5593	100	0.001534006	OTU_797	100	0.001432946
OTU_1501	100	0.000239829	OTU_4329	100	0.00157624	OTU_4619	100	0.00370605	OTU_2848	100	0.001911851
OTU_1545	100	4.98E-05	OTU_4328	100	0.001470646	OTU_4970	100	0.001325852	OTU_865	100	0.000644833
OTU_165	100	8.15E-05	OTU_747	100	0.002043833	OTU_4214	100	0.002891534	OTU_894	100	0.000260201
OTU_171	100	0.000366537	OTU_4325	100	0.001229312	OTU_809	100	0.003434545	OTU_954	100	0.001610178
OTU_1734	100	0.003420964	OTU_3696	100	0.001161436	OTU_4625	100	0.0037151	OTU_3690	100	0.00194956
OTU_1740	100	5.88E-05	OTU_2784	100	0.00120292	OTU_4316	100	0.001791936	OTU_4034	100	0.00107848
OTU_1753	100	0.000176474	OTU_3698	100	0.001399008	OTU_5348	100	0.002081542	OTU_3685	100	0.001972186
OTU_1784	100	4.53E-05	OTU_4970	100	0.00110488	OTU_5322	100	0.001108647	OTU_15	100	0.001157665
OTU_1799	100	0.000561111	OTU_3681	100	0.001346215	OTU_941	100	0.001217244	OTU_4325	100	0.001289651
OTU_18	100	0.00050681	OTU_4848	100	0.000429888	OTU_3696	100	0.001167474	OTU_3381	100	0.000984208
OTU_1831	100	0.003420969	OTU_4317	100	0.000814516	OTU_6034	100	0.000855242	OTU_1110	100	0.000584495
OTU_1875	100	2.26E-05	OTU_797	100	0.000437429	OTU_869	100	0.00126251	OTU_5667	100	0.001074705
OTU_2038	100	0.000257925	OTU_875	100	0.000825825	OTU_529	100	0.003262596	OTU_699	100	0.000822067

OTU_2102	100	0.002307797	OTU_4339	100	0.00122177	OTU_4393	100	0.00047966	OTU_18	100	0.001293422
OTU_2194	100	0.000276035	OTU_4389	100	0.000667455	OTU_1831	100	0.002321372	OTU_3724	100	0.001425404
OTU_2195	100	0.000692339	OTU_4331	100	0.00146688	OTU_3666	100	0.002371148	OTU_4312	100	0.001067167
OTU_2219	100	0.000343906	OTU_971	100	0.000467601	OTU_1356	100	0.00171048	OTU_117	100	0.000241363
OTU_2302	100	0.000289606	OTU_4891	100	0.000829608	OTU_4317	100	0.000280551	OTU_813	100	0.001150144
OTU_2389	100	0.000466099	OTU_3699	100	0.000716472	OTU_5650	100	0.000733065	OTU_5954	100	0.0012444
OTU_2414	100	4.53E-05	OTU_5954	100	0.001040771	OTU_797	100	0.001629028	OTU_4411	100	0.000312981
OTU_2546	100	0.00172859	OTU_3497	100	0.001112418	OTU_2848	100	0.001022671	OTU_3497	100	0.000889935
OTU_2690	100	0.000900488	OTU_3630	100	0.001327356	OTU_3679	100	0.000850717	OTU_121	100	4.15E-05
OTU_2723	100	0.000348422	OTU_3684	100	0.001123723	OTU_5337	100	0.000886918	OTU_5508	100	0.000656146
OTU_2783	100	0.003058962	OTU_3584	100	0.001244408	OTU_865	100	0.002466175	OTU_2690	100	0.000312989
OTU_2786	100	0.000104077	OTU_3381	100	0.000931415	OTU_894	100	0.002846283	OTU_4341	100	0.00117654
OTU_2801	100	0.000343912	OTU_1110	100	0.000150836	OTU_1026	100	0.002493335	OTU_3566	100	0.001429175
OTU_2815	100	0.000190068	OTU_5340	100	0.00117652	OTU_954	100	0.001117702	OTU_4848	100	0.000509077
OTU_2837	100	8.60E-05	OTU_1353	100	0.000799433	OTU_3472	100	0.002226345	OTU_4718	100	0.000720244
OTU_2838	100	0.000407263	OTU_952	100	0.00044875	OTU_3341	100	0.002081542	OTU_948	100	0.001127502

OTU_2848	100	0.00074664	OTU_4718	100	0.000230017	OTU_4034	100	0.001529481	OTU_4812	100	0.001037012
OTU_2857	100	9.95E-05	OTU_5235	100	0.000460063	OTU_5085	100	0.002647178	OTU_4434	100	0.000437429
OTU_2869	100	9.96E-05	OTU_4393	100	0.000855992	OTU_826	100	0.000574676	OTU_3825	100	0.001048313
OTU_2981	100	0.000149323	OTU_5642	100	0.000369553	OTU_3685	100	0.000348432	OTU_3698	100	0.000712702
OTU_30	100	0.013747228	OTU_865	100	0.000384649	OTU_3699	100	0.001524956	OTU_4331	100	0.000426121
OTU_3177	100	0.000420833	OTU_139	100	0.001153881	OTU_866	100	0.00223542	OTU_3584	100	0.001116181
OTU_3323	100	5.89E-05	OTU_5336	100	0.000693855	OTU_4325	100	0.000914069	OTU_5351	100	0.000301672
OTU_3328	100	0.007525227	OTU_3678	100	0.000576945	OTU_3381	100	0.001280601	OTU_4322	100	0.000675001
OTU_3341	100	0.000991	OTU_4489	100	0.00089371	OTU_3409	100	0.002063442	OTU_3684	100	0.001021929
OTU_3381	100	0.000420833	OTU_813	100	0.00054681	OTU_1297	100	0.002027251	OTU_3695	100	0.000663684
OTU_3409	100	0.002538576	OTU_5956	100	0.000720244	OTU_1110	100	0.001701434	OTU_5956	100	0.000901251
OTU_3459	100	0.001868857	OTU_3695	100	0.000426117	OTU_5667	100	0.001104117	OTU_989	100	0.000139536
OTU_3472	100	0.001810046	OTU_122	100	0.001014387	OTU_928	100	0.002054387	OTU_5340	100	0.000652371
OTU_3497	100	0.000438939	OTU_1001	100	0.000561865	OTU_1023	100	0.00222182	OTU_3873	100	0.000848455
OTU_3579	100	0.000294125	OTU_5356	100	0.000588262	OTU_699	100	0.001325852	OTU_5549	100	0.000475147
OTU_3630	100	0.000117657	OTU_6034	100	0.000686306	OTU_18	100	0.000683289	OTU_531	100	0.000554324

OTU_3660	100	0.03187022	OTU_2848	100	0.000324298	OTU_3724	100	0.000524911	OTU_971	100	0.000625971
OTU_3661	100	0.004407439	OTU_4312	100	0.000682535	OTU_969	100	0.001357532	OTU_5235	100	0.000614662
OTU_3662	100	0.010018553	OTU_5339	100	0.000644837	OTU_4312	100	0.000886913	OTU_4900	100	0.000799433
OTU_3666	100	0.003380243	OTU_1019	100	0.000377099	OTU_943	100	0.001674279	OTU_3742	100	0.000576949
OTU_3678	100	0.000701389	OTU_5621	100	0.00065993	OTU_117	100	0.001855282	OTU_4909	100	0.000686305
OTU_3679	100	0.000796421	OTU_5355	100	0.000196103	OTU_5954	100	0.000642563	OTU_5336	100	0.000543019
OTU_3681	100	0.000615417	OTU_3742	100	0.000592029	OTU_3497	100	0.001049831	OTU_1011	100	0.000196091
OTU_3684	100	0.000334871	OTU_1491	100	0.000309235	OTU_1734	100	0.001823612	OTU_1001	100	0.000362015
OTU_3685	100	0.000538491	OTU_4441	100	0.000290372	OTU_4050	100	0.00121725	OTU_2814	100	0.000418567
OTU_3695	100	0.000859767	OTU_3753	100	0.000365774	OTU_1309	100	0.00173311	OTU_5958	100	0.000543019
OTU_3696	100	0.001063396	OTU_4659	100	0.000218712	OTU_3678	100	0.000199114	OTU_3717	100	0.000426113
OTU_3698	100	0.000642563	OTU_5353	100	0.000558095	OTU_3680	100	0.000678768	OTU_123	100	0.000441213
OTU_3699	100	0.000932169	OTU_15	100	0.000169682	OTU_5508	100	0.001158423	OTU_5955	100	0.000576949
OTU_3702	100	0.000543	OTU_5562	100	0.000433666	OTU_2389	100	0.001868858	OTU_4441	100	0.000350703
OTU_3709	100	0.000122192	OTU_4827	100	0.000441213	OTU_4341	100	0.000466079	OTU_5353	100	0.000490214
OTU_3710	100	0.000289625	OTU_5392	100	0.000354466	OTU_1334	100	0.001710484	OTU_5349	100	0.000267739

OTU_3711	100	0.000850732	OTU_4404	100	0.000546782	OTU_1397	100	0.001479714	OTU_5957	100	0.000384641
OTU_3714	100	0.000285075	OTU_3701	100	0.000527927	OTU_4389	100	0.000660663	OTU_153	100	0.000573187
OTU_3716	100	0.00024888	OTU_153	100	0.000569411	OTU_4718	100	0.000814516	OTU_5339	100	0.000444967
OTU_3719	100	0.000230779	OTU_3717	100	0.000260192	OTU_692	100	0.001438974	OTU_4814	100	0.000584507
OTU_3721	100	0.000389167	OTU_5955	100	0.000490222	OTU_948	100	0.000239834	OTU_5428	100	0.000362007
OTU_3723	100	0.000239829	OTU_5361	100	0.000331856	OTU_4812	100	0.000334851	OTU_5642	100	0.000275284
OTU_3724	100	0.000877878	OTU_5959	100	0.000501531	OTU_4434	100	0.00101815	OTU_975	100	4.53E-05
OTU_3732	100	9.50E-05	OTU_1123	100	0.000203629	OTU_3825	100	0.000285091	OTU_3753	100	0.000426117
OTU_3737	100	0.000470619	OTU_531	100	0.000282827	OTU_1387	100	0.001230825	OTU_4323	100	0.000377095
OTU_3742	100	0.000303181	OTU_3763	100	0.000256422	OTU_2801	100	0.001140323	OTU_3462	100	0.000377095
OTU_3744	100	9.95E-05	OTU_3873	100	0.000294131	OTU_3698	100	0.000651613	OTU_5333	100	0.000147078
OTU_3757	100	9.95E-05	OTU_3516	100	0.000448738	OTU_3584	100	0.000108592	OTU_5366	100	0.000173466
OTU_3758	100	0.000325821	OTU_5366	100	0.000286589	OTU_3459	100	0.001158423	OTU_4119	100	0.000101826
OTU_3759	100	0.000230779	OTU_4814	100	0.000448729	OTU_4460	100	0.000601842	OTU_3714	100	0.000252663
OTU_3795	100	9.96E-05	OTU_5401	100	0.000173462	OTU_5351	100	0.001054346	OTU_1002	100	0.000290368
OTU_3798	100	7.70E-05	OTU_3347	100	0.000399724	OTU_4322	100	0.000601832	OTU_4314	100	0.000154611

OTU_3809	100	0.000122177	OTU_3888	100	0.000275277	OTU_3695	100	0.000588262	OTU_1123	100	0.000358236
OTU_3828	100	0.000167442	OTU_3883	100	0.000241342	OTU_3691	100	0.000411788	OTU_3710	100	0.000418583
OTU_3842	100	4.07E-05	OTU_118	100	0.000218721	OTU_5956	100	0.000294131	OTU_3516	100	0.000297902
OTU_3854	100	4.53E-05	OTU_5354	100	0.000188557	OTU_989	100	0.001203674	OTU_110	100	0.000335603
OTU_3863	100	0.017335626	OTU_5349	100	0.000226258	OTU_5340	100	0.000565626	OTU_3718	100	0.00024888
OTU_3883	100	0.000289601	OTU_4411	100	0.00011691	OTU_3873	100	0.000298666	OTU_5621	100	0.000252651
OTU_3960	100	0.006751437	OTU_948	100	0.000233809	OTU_1478	100	0.001257976	OTU_4439	100	8.30E-05
OTU_3986	100	7.24E-05	OTU_5344	100	0.000162157	OTU_785	100	0.00122177	OTU_5356	100	0.000301681
OTU_3997	100	0.000375597	OTU_3690	100	0.000222471	OTU_5549	100	0.000705914	OTU_4404	100	0.000263968
OTU_4016	100	0.000126707	OTU_5394	100	0.000184778	OTU_4361	100	0.000683284	OTU_4830	100	0.000165919
OTU_4028	100	8.60E-05	OTU_2569	100	0.000230017	OTU_531	100	0.000565641	OTU_2838	100	8.68E-05
OTU_4050	100	0.001081492	OTU_3823	100	0.000177232	OTU_971	100	0.000470604	OTU_3702	100	0.000373332
OTU_4073	100	0.000208159	OTU_4323	100	0.000214946	OTU_1799	100	0.001140323	OTU_3781	100	0.000188541
OTU_4214	100	0.003384768	OTU_5350	100	0.000180999	OTU_4313	100	0.000398217	OTU_4413	100	0.000252646
OTU_4311	100	0.0010272	OTU_5405	100	0.000124448	OTU_4900	100	0.000217214	OTU_3888	100	0.000199862
OTU_4312	100	0.000271505	OTU_3747	100	0.000233796	OTU_2786	100	8.15E-05	OTU_3883	100	0.000260189

OTU_4313	100	0.00028509	OTU_5413	100	0.00022625	OTU_3177	100	0.000769266	OTU_3747	100	0.000271509
OTU_4314	100	0.004366713	OTU_1055	100	0.000165916	OTU_963	100	0.000778315	OTU_3576	100	0.000252655
OTU_4316	100	0.004461745	OTU_3323	100	0.000230021	OTU_940	100	0.000484184	OTU_5401	100	0.000143307
OTU_4317	100	0.001217249	OTU_3482	100	0.000154595	OTU_3457	100	0.0005023	OTU_5959	100	0.000290356
OTU_4318	100	0.000615412	OTU_3576	100	0.000218729	OTU_1154	100	0.000855247	OTU_165	100	0.000147057
OTU_4319	100	0.004099733	OTU_3759	100	6.79E-05	OTU_3742	100	0.000393683	OTU_5431	100	0.000124432
OTU_4320	100	0.000203634	OTU_5424	100	0.000173449	OTU_4909	100	0.000257935	OTU_4543	100	0.000279038
OTU_4322	100	0.000533961	OTU_4829	100	0.000147082	OTU_3711	100	0.000393692	OTU_5389	100	0.000188541
OTU_4324	100	0.002063442	OTU_5852	100	0.000181012	OTU_949	100	0.000805476	OTU_5200	100	0.000173466
OTU_4325	100	0.000981949	OTU_5438	100	0.000135752	OTU_2723	100	0.000325807	OTU_1054	100	0.000139523
OTU_4326	100	0.000583742	OTU_4361	100	0.000135765	OTU_4929	100	0.000787361	OTU_4350	100	0.000214946
OTU_4329	100	0.000877868	OTU_5397	100	6.41E-05	OTU_1001	100	0.000552066	OTU_3750	100	0.00020742
OTU_4339	100	0.000660658	OTU_3820	100	0.000124448	OTU_120	100	0.000918588	OTU_4867	100	0.000162145
OTU_4341	100	0.000325811	OTU_4028	100	0.000131985	OTU_5562	100	0.000638042	OTU_5387	100	0.000120681
OTU_4350	100	0.000180999	OTU_4806	100	0.000124443	OTU_994	100	0.000696864	OTU_4352	100	0.000135748
OTU_4357	100	0.000153873	OTU_5387	100	9.80E-05	OTU_4339	100	0.000348432	OTU_4885	100	0.000158378

OTU_4364	100	0.000113167	OTU_148	100	0.000143286	OTU_3630	100	0.000321276	OTU_2829	100	0.000199866
OTU_4376	100	6.79E-05	OTU_5966	100	0.000158374	OTU_5362	100	0.00025341	OTU_5451	100	0.000120681
OTU_4377	100	0.067310738	OTU_5620	100	0.000131981	OTU_953	100	0.000552061	OTU_5413	100	0.000158378
OTU_4380	100	0.003321417	OTU_5719	100	9.81E-05	OTU_5958	100	0.0002353	OTU_4532	100	6.03E-05
OTU_4382	100	0.000339372	OTU_4830	100	7.16E-05	OTU_5350	100	0.000221729	OTU_5354	100	8.30E-05
OTU_4389	100	0.001185579	OTU_5995	100	7.17E-05	OTU_5480	100	0.000624462	OTU_5995	100	0.000181003
OTU_4390	100	0.033196082	OTU_4932	100	0.000105585	OTU_2306	100	0.000737585	OTU_5965	100	0.000173466
OTU_4393	100	0.000398203	OTU_210	100	4.90E-05	OTU_3717	100	0.000316756	OTU_5442	100	0.00013954
OTU_4413	100	0.000113127	OTU_2879	100	9.05E-05	OTU_4659	100	0.000574687	OTU_4880	100	7.54E-05
OTU_4434	100	0.000547536	OTU_4773	100	4.90E-05	OTU_5955	100	0.000122187	OTU_4829	100	0.000128223
OTU_4439	100	0.00051586	OTU_3663	100	8.30E-05	OTU_4441	100	0.000389158	OTU_4967	100	0.000150853
OTU_4441	100	0.000547536	OTU_5009	100	5.66E-05	OTU_686	100	0.000352967	OTU_5620	100	0.000113123
OTU_4446	100	0.000158383	OTU_50	100	7.17E-05	OTU_1353	100	0.000610887	OTU_3776	100	0.00010181
OTU_4504	100	0.000253424	OTU_4353	100	6.03E-05	OTU_5359	100	0.000375582	OTU_4845	100	0.000139515
OTU_4532	100	0.000389153	OTU_3592	100	6.41E-05	OTU_5346	100	4.52E-05	OTU_5961	100	0.000124439
OTU_4543	100	7.24E-05				OTU_4827	100	0.000285076	OTU_4806	100	0.000101814

OTU_4564	100	0.002013666	OTU_4871	100	0.000217214	OTU_4904	100	7.54E-05
OTU_4574	100	0.000226269	OTU_5349	100	0.000438939	OTU_4630	100	7.17E-05
OTU_4619	100	0.001434454	OTU_4824	100	0.000429879	OTU_534	100	8.67E-05
OTU_4621	100	0.000217219	OTU_5957	100	0.000294145	OTU_5447	100	6.79E-05
OTU_4625	100	0.003249016	OTU_5339	100	0.000212674	OTU_4028	100	4.90E-05
OTU_4648	100	0.000185538	OTU_5428	100	0.000285091	OTU_5964	100	6.04E-05
OTU_4659	100	0.000615417	OTU_4836	100	0.000407268	OTU_5764	100	6.03E-05
OTU_4718	100	0.001181049	OTU_975	100	0.000651613	OTU_5004	100	5.28E-05
OTU_4811	100	0.000945734	OTU_3753	100	0.000190058			
OTU_4812	100	0.000466079	OTU_4328	100	0.000538491			
OTU_4815	100	0.000135767	OTU_220	100	0.000407253			
OTU_4827	100	0.000325821	OTU_3462	100	0.000230789			
OTU_4829	100	0.000131212	OTU_4375	100	0.000357481			
OTU_4831	100	8.60E-05	OTU_5381	100	0.000434418			
OTU_4883	100	0.004773972	OTU_5366	100	0.000457034			
OTU_4900	100	0.000221739	OTU_1059	100	0.00048419			

OTU_4968	100	0.000140292	OTU_1209	100	0.000294131
OTU_4970	100	0.000945744	OTU_1076	100	0.000565641
OTU_4975	100	9.05E-05	OTU_1002	100	0.000298656
OTU_5046	100	9.05E-05	OTU_3482	100	0.000393688
OTU_5085	100	0.004086168	OTU_67	100	0.000515855
OTU_5107	100	0.000176473	OTU_5397	100	0.000357487
OTU_5200	100	0.00025341	OTU_1123	100	0.000181003
OTU_531	100	0.000357492	OTU_4574	100	0.000511325
OTU_5322	100	0.001366578	OTU_2833	100	0.000556596
OTU_5337	100	0.000457034	OTU_5392	100	0.000235304
OTU_5344	100	0.00025793	OTU_5365	100	0.000425368
OTU_5348	100	0.001941264	OTU_5363	100	0.000533971
OTU_5350	100	0.000194594	OTU_110	100	0.000167433
OTU_5351	100	0.000778311	OTU_127	100	0.00028962
OTU_5354	100	0.000262469	OTU_2327	100	0.000497759
OTU_5355	100	0.000787361	OTU_3718	100	0.00026245

OTU_5356	100	0.000647087	OTU_4811	100	0.000506815
OTU_5359	100	0.000212674	OTU_3347	100	0.000153853
OTU_5361	100	0.000339387	OTU_4933	100	0.000334856
OTU_5366	100	0.000303191	OTU_1394	100	0.0005023
OTU_5369	100	0.000217199	OTU_2369	100	0.000375592
OTU_5372	100	0.000941229	OTU_5356	100	0.000171953
OTU_5380	100	0.000321281	OTU_4404	100	0.000212674
OTU_5383	100	0.000339381	OTU_1036	100	0.000506805
OTU_5390	100	0.000687804	OTU_5385	100	0.00029413
OTU_5392	100	0.000411783	OTU_2781	100	0.000348442
OTU_5394	100	0.000221724	OTU_332	100	0.000389157
OTU_5397	100	0.000203629	OTU_4320	100	0.000461559
OTU_5398	100	0.00023531	OTU_1491	100	0.000307716
OTU_5409	100	0.00053848	OTU_2302	100	0.000366533
OTU_5413	100	9.05E-05	OTU_3702	100	3.62E-05
OTU_5417	100	0.000475134	OTU_3781	100	0.00024888

OTU_5428	100	0.000398213
OTU_5430	100	0.000104077
OTU_5438	100	0.000140282
OTU_5442	100	0.000140282
OTU_5444	100	0.000316761
OTU_5449	100	0.000185538
OTU_5450	100	0.000149348
OTU_5464	100	4.98E-05
OTU_5480	100	0.001579261
OTU_5508	100	0.0024707
OTU_5533	100	0.000176508
OTU_5621	100	0.000248875
OTU_5642	100	0.00097742
OTU_5667	100	0.000696864
OTU_5852	100	8.60E-05
OTU_5954	100	0.000533961
OTU_3758	100	0.000181023
OTU_3583	100	0.000230779
OTU_3888	100	0.000221734
OTU_1665	100	0.000434398
OTU_3883	100	0.000144798
OTU_3850	100	4.98E-05
OTU_5394	100	0.000298661
OTU_2219	100	0.000380102
OTU_3747	100	0.000108612
OTU_5401	100	0.00025341
OTU_3490	100	0.000334862
OTU_5372	100	0.00026699
OTU_1018	100	0.000226244
OTU_1317	100	0.000199108
OTU_1205	100	0.000262455
OTU_5422	100	0.00029414

OTU_5955	100	0.000167433	OTU_5431	100	0.000226249
OTU_5956	100	0.00051134	OTU_4920	100	0.000104091
OTU_5958	100	0.000303171	OTU_1308	100	0.000312231
OTU_5973	100	9.96E-05	OTU_3708	100	0.000343907
OTU_6034	100	0.000416318	OTU_593	100	0.000190059
OTU_63	100	0.001312276	OTU_997	100	0.00026246
OTU_643	100	0.010389615	OTU_2827	100	0.000162908
OTU_67	100	0.001325847	OTU_3729	100	0.000113137
OTU_686	100	0.000543021	OTU_3737	100	0.000126702
OTU_699	100	0.00124441	OTU_5200	100	0.000140282
OTU_704	100	0.016647812	OTU_3716	100	0.000208159
OTU_759	100	0.009982352	OTU_4384	100	0.000199094
OTU_764	100	0.005059053	OTU_5371	100	0.000334847
OTU_777	100	0.008344269	OTU_5370	100	0.000144812
OTU_809	100	0.006602119	OTU_4894	100	5.43E-05
OTU_813	100	0.000719489	OTU_3795	100	0.000149338

OTU_825	100	0.005869044	OTU_5387	100	0.000162912
OTU_865	100	0.000945739	OTU_4881	100	0.000144822
OTU_874	100	0.00693244	OTU_4885	100	0.000108622
OTU_875	100	0.001135798	OTU_5449	100	0.000181028
OTU_877	100	0.000208149	OTU_2829	100	5.43E-05
OTU_894	100	0.004416489	OTU_4504	100	0.000221744
OTU_895	100	0.004733246	OTU_1240	100	0.000226249
OTU_925	100	0.001488755	OTU_1302	100	0.000140287
OTU_926	100	0.001212724	OTU_5451	100	0.000140292
OTU_928	100	0.008122549	OTU_33	100	0.000190068
OTU_937	100	0.000325806	OTU_1486	100	0.000226274
OTU_938	100	0.006918865	OTU_5255	100	8.14E-05
OTU_941	100	0.00271958	OTU_3407	100	8.60E-05
OTU_946	100	0.001905064	OTU_154	100	5.88E-05
OTU_948	100	0.000181012	OTU_3508	100	0.000122182
OTU_953	100	0.000687814	OTU_4532	100	0.000185528

OTU_954	100	0.00225803	OTU_1301	100	0.000171958
OTU_969	100	0.001122237	OTU_3323	100	6.34E-05
OTU_971	100	0.001398253	OTU_176	100	0.000104086
OTU_986	100	0.000230784	OTU_1257	100	0.000235304
OTU_988	100	0.000307696	OTU_5973	100	9.05E-05
OTU_989	100	0.000167423	OTU_2931	100	0.000108616
OTU_994	100	0.000438934	OTU_3732	100	7.24E-05
OTU_997	100	0.000321286	OTU_1226	100	0.000181013
			OTU_1501	100	0.000171943
			OTU_1355	100	0.000208149
			OTU_4364	100	7.24E-05
			OTU_1261	100	0.000122177
			OTU_2857	100	0.000131232
			OTU_5430	100	0.000149338
			OTU_4968	100	5.43E-05
			OTU_2038	100	0.000190053

OTU_5442	100	5.43E-05
OTU_4829	100	6.34E-05
OTU_1083	100	0.000153863
OTU_5426	100	9.05E-05
OTU_1000	100	6.34E-05
OTU_4815	100	0.000126697
OTU_5620	100	7.24E-05
OTU_4461	100	0.000149343
OTU_5406	100	4.98E-05
OTU_242	100	0.000117652
OTU_3824	100	9.50E-05
OTU_2010	100	0.000181003
OTU_5380	100	0.000108587
OTU_1753	100	6.79E-05
OTU_3719	100	0.000113137
OTU_1630	100	0.000135747

OTU_1096	100	9.50E-05
OTU_137	100	0.000158383
OTU_5716	100	0.000167423
OTU_5852	100	9.96E-05
OTU_3723	100	8.60E-05
OTU_4870	100	6.79E-05
OTU_1157	100	5.88E-05
OTU_1369	100	9.96E-05
OTU_3745	100	7.24E-05
OTU_3525	100	0.000113137
OTU_5867	100	0.000158393
OTU_3693	100	0.000135752
OTU_5070	100	9.50E-05
OTU_4605	100	0.000104081
OTU_277	100	0.000117692
OTU_1084	100	0.000108606

OTU_1638	100	8.60E-05
OTU_5450	100	9.05E-05
OTU_2946	100	0.000108602
OTU_1522	100	9.05E-05
OTU_5429	100	6.79E-05
OTU_3827	100	5.43E-05
OTU_3986	100	2.71E-05
OTU_3586	100	0.000117652
OTU_4882	100	5.88E-05
OTU_1782	100	6.79E-05
OTU_4371	100	4.98E-05
OTU_4646	100	5.43E-05
OTU_3269	100	3.62E-05
OTU_1786	100	0.000104086
OTU_3818	100	5.43E-05
OTU_193	100	4.08E-05

OTU_2204	100	6.33E-05
OTU_1765	100	6.79E-05
OTU_1552	100	6.34E-05
OTU_164	100	5.43E-05
OTU_5444	100	7.24E-05
OTU_4538	100	4.98E-05
OTU_2198	100	5.43E-05
OTU_288	100	5.43E-05
OTU_350	100	4.53E-05
OTU_5227	100	3.17E-05
OTU_3782	100	2.71E-05
OTU_2353	100	4.52E-05
OTU_567	100	4.07E-05
OTU_2301	100	4.53E-05
OTU_5579	100	3.62E-05
OTU_1732	100	2.71E-05

OTU_2214	100	2.26E-05
