# Supplementary material for: Association between weaning stress and rumen microbiota in goat kids: evidence from granger causality and randomized controlled trial validation
Source: Anim Biosci. 2025 Aug 25;39(1):250092. doi: 10.5713/ab.25.0092 (PMC12754500; doi:10.5713/ab.25.0092)
Supplement: Supplementary file 6 [file ab-25-0092-Supplementary-6.pdf]

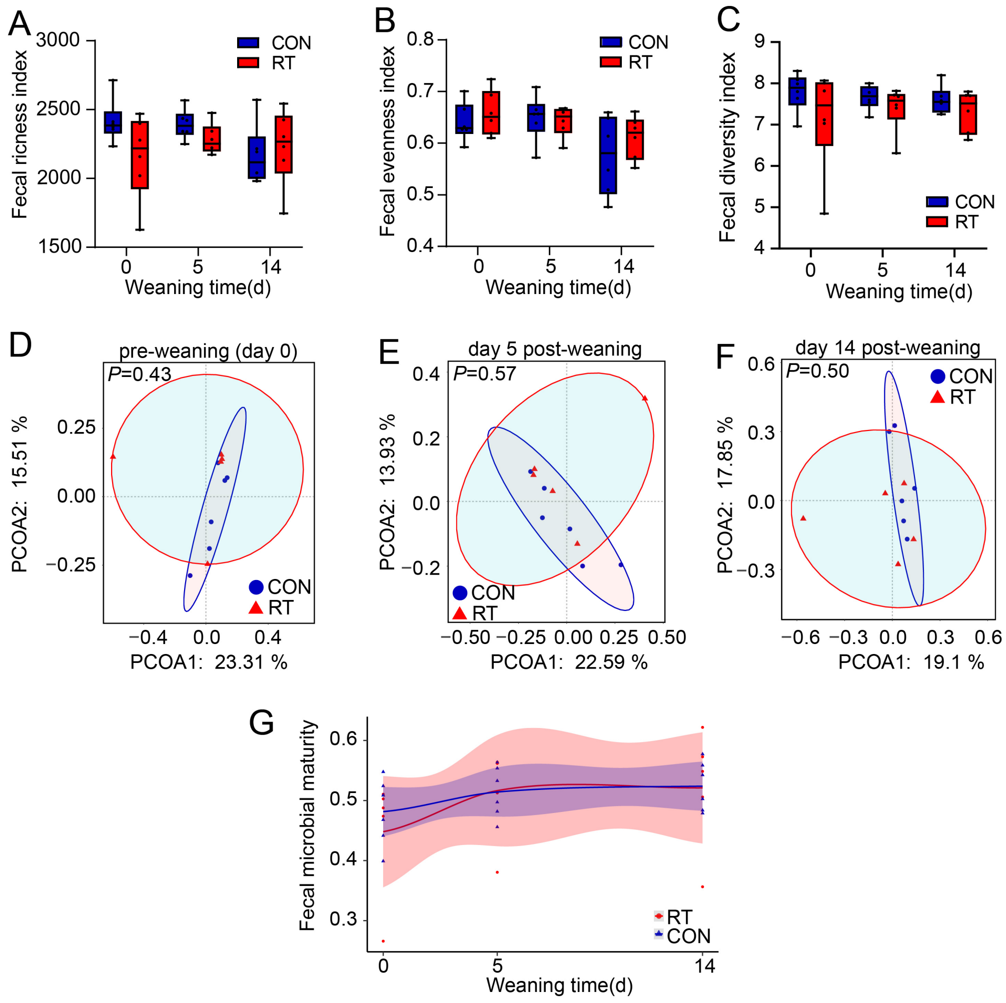

Supplement 6. The effect of cultured rumen mcrobiota transplantation on the intestinal bacterial communities of goat kids. Richness index (A), Evenness index (B), and Diversity index (C) of intestinal microbiota between groups; Principal coordinate analysis (PCoA) of gut microbiota at the OTU level at pre-weaning (0 days, D), 5 days(E), and 14 days (F) post-weaning. (G) The maturity of intestinal microbiota in the RT and CON groups.
